# Recent advances in polymeric microparticle-based drug delivery systems for knee osteoarthritis treatment

**DOI:** 10.3389/fbioe.2023.1290870

**Published:** 2023-12-07

**Authors:** Guangxin Wang, Xin-an Zhang, Leonid Kapilevich, Mingjie Hu

**Affiliations:** ^1^ Department of Orthopedics, The Fourth People’s Hospital of Shenyang, Shenyang, China; ^2^ College of Exercise and Health, Shenyang Sport University, Shenyang, China; ^3^ Faculty of Physical Education, Nаtionаl Reseаrch Tomsk Stаte University, Tomsk, Russiа

**Keywords:** knee osteoarthritis, drug delivery systems, intra-articular, microparticles, biodegradable polymers

## Abstract

Due to the poor bioavailability and high joint clearance of drugs, sustained delivery of therapeutic agents has proven difficult in the treatment of osteoarthritis (OA). Intra-articular (IA) drug delivery strategy is an attractive option for enhancing OA patients’ prognosis, for which various polymer materials have been used as drug carriers due to their attractive delivery properties, to slow or even reverse the progress of OA by prolonging the duration of therapeutic agent residence in the joint. This article focuses on the recent developments in natural and synthetic polymer-based microsphere drug delivery systems for treating knee osteoarthritis. It evaluates the translational potential of some novel formulations for clinical application.

## 1 Introduction

Osteoarthritis (OA) is the most common degenerative disease of the joints and is characterized by cartilage degeneration and bone hyperplasia, and is emerging as a major cause of chronic disability (He et al., 2017; Cucchiarini and Madry, 2019), influencing over 300 million individuals globally (Boer et al., 2021). Although OA may be genetic, there are many risk factors associated with its development; among them, aging, gender, and obesity are the direct influences (Ondresik et al., 2017), and aging is a major risk factor for the OA progression, which impacts both the mechanical and biochemical changes within tissue’s structure (Bellamy et al., 1997; Bas et al., 2018; Berenbaum et al., 2018; Peters et al., 2018); and female cartilage is thinner, leading to greater cartilage wear and tear, and there are larger differences in the mechanical alignment (Blanco and Rego-Pérez, 2018), they are more susceptible to OA than men; Obesity can limit physical activity and lead to muscle weakness, which can also greatly increase the prevalence of OA; in addition, excessive mechanical load, strenuous physical activity, and insufficient nutritional supply are also several important factors that lead to joint degeneration (Bajpayee and Grodzinsky, 2017; Abbas et al., 2018; Ahmad et al., 2019; Jain & Ravikumar, 2020).

Knee osteoarthritis (KOA) is a chronic joint disease involving the entire knee joint (Johnson and Hunter, 2014; Glyn-Jones et al., 2015; Park et al., 2021), and is common in middle-aged and elderly adults (Jamshidi et al., 2019; Sharma, 2021). The prevalence of KOA has more than doubled in the last decade (Palazzo et al., 2016; Kyu et al., 2018), accounting for about 85% of all OA cases worldwide (Vos et al., 2016; James et al., 2018), making it an increasingly important public health challenge in the coming decades.

KOA is characterised by dysfunction of the knee joint and persistent pain. It is caused by joint deformation and destruction (Eitner et al., 2017). Gradual cartilage erosion stimulates chondrocytes to enhance anabolism via compensatory hypertrophy (Goldring and Goldring, 2016), which generates both degradation products and pro-inflammatory factors to expedite KOA development (Hunter and Bierma-Zeinstra, 2019). Therefore, early treatment after the disease is advocated to reduce the inflammatory response and delay the joints degeneration (Fan et al., 2018; Törmälehto et al., 2018). The articular cartilage of KOA patients usually degenerates gradually. The degenerated cartilage becomes worn and rough (Lane and Corr, 2017), resulting in symptoms like muscle weakness, pain and stiffness in the knee joint, which in the long run can lead to reduced physical activity, decreased physical function, sleep disturbance, fatigue, depression, and even disability.

Currently, the diagnosis of OA is usually made by X-ray imaging, MRI, or joint fluid analysis after the patient’s medical history and physical examination (Braun and Gold, 2012; Demehri et al., 2016). Osteoarthritis cannot be cured entirely at present, and most treatment options rely solely on symptomatic interventions, with a particular focus on relieving pain and enhancing physical function. The treatment depends largely on the severity of OA and the patient’s degree of pain, and there are no disease-relieving therapies to stabilize or reverse the progression of OA. The first choice is the treatment of symptomatic pain, with analgesics, specific cyclooxygenase-2 (COX-2) inhibitors, non-steroidal anti-inflammatory drugs (NSAIDs), and opioids commonly used for systemic treatment (Block et al., 2010; Bowman et al., 2018; da Costa et al., 2017; Lane, 1997; Leopoldino et al., 1996). However, many traditional medicines have significant risk profiles. They may cause gut, heart, or brain side-effects. As OA treatments evolve, intra-articular (IA) injections have become an option alongside oral and local treatments (Nelson et al., 2014). In contrast, IA can effectively avoid systemic toxicities (Nguyen and Rannou, 2017) and improve the drug bioavailability, thus reducing the cost of treatment. Also, IA is the last option before hip or knee replacement surgery.

Nevertheless, intra-articular injection remains an invasive therapy, and the quick elimination of small-molecule drugs in the joint is one of the main limitations of the current clinical methods for the treatment of osteoarthritis. This means that most drugs remain in the joint for only several hours after injection before being rapidly eliminated because of the joint’s unique physiological environment (Owen et al., 1994; Schumacher, 2003; Gerwin et al., 2006; Chevalier et al., 2009; Vugmeyster et al., 2012). As a result, repeated intra-articular injections are necessary, which will greatly reduce patient adherence and increase the risk of infection (Lin et al., 2004). In addition, IA only relieves inflammatory symptoms to slow the disease’s advancement, but reversing the disease course to successfully treat OA is difficult.

Therefore, ensuring continuous and effective drug delivery to joint targets remains a major challenge (Evans et al., 2014), and advanced drug delivery strategies hold promise for improving OA patient outcomes by prolonging the release cycle of medicinal substances in the knee joint. The encapsulation of drugs in polymeric drug delivery systems (DDSs) for controlled release is the fundamental principle of this strategy. The goal is the direct delivery of the therapeutic agent to the targeted tissue to increase the drug concentration in the affected area, thereby reducing the dosage necessary to produce the desired therapeutic result. To prolong drug residence time in joints, reduce dosing frequency and side effects, researchers have worked to develop DDSs with sustained release effects (Zhang et al., 2018; Kou et al., 2019; Xue et al., 2021).

Among them, the microsphere delivery systems may provide a potential way to improve the co-retention of small molecule drugs. Polymeric microspheres range in size from approximately 1–100 µm (Bhaskar et al., 2010; He and Park, 2016). Microspheres can generally be prepared from natural or synthetic polymers acting as matrices. In this review, we outline current developments in knee intra-articular drug delivery systems in recent years and evaluate the application prospects of microscale DDSs based on different carrier matrices for KOA therapy.

## 2 Application of polymeric microparticles (MPs) in knee osteoarthritis treatment

### 2.1 Natural polymeric microparticle-based drug delivery systems

**FIGURE 1:**
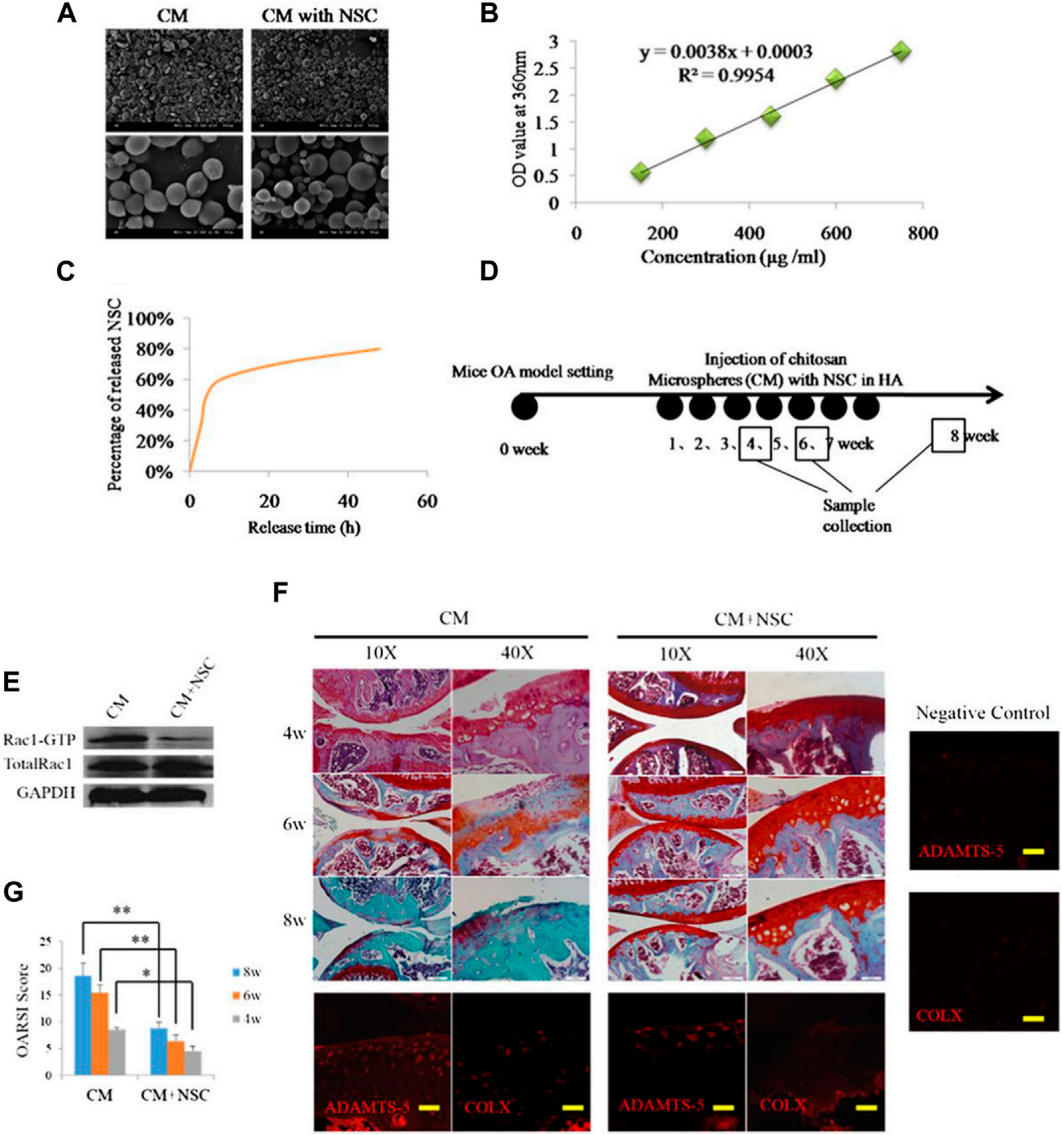
**(A)** SEM images of CS microspheres. **(B)** Standard curve of NSC23766. **(C)** Release curve of CS microspheres. **(D)** Animal study schedule. **(E)** The inhibitory effect of release of NSC23766 on Rac1 activity in chondrocytes. **(F)** Results of safranin staining and gene expression. **(G)** OARSI scoring of OA severity following treatment with HA containing NSC23766 loaded CS microspheres. Reproduced with permission from ref (Zhu et al., 2015). CC BY-NC 3.0. Copyright 2015 The Author(s).

**FIGURE 2:**
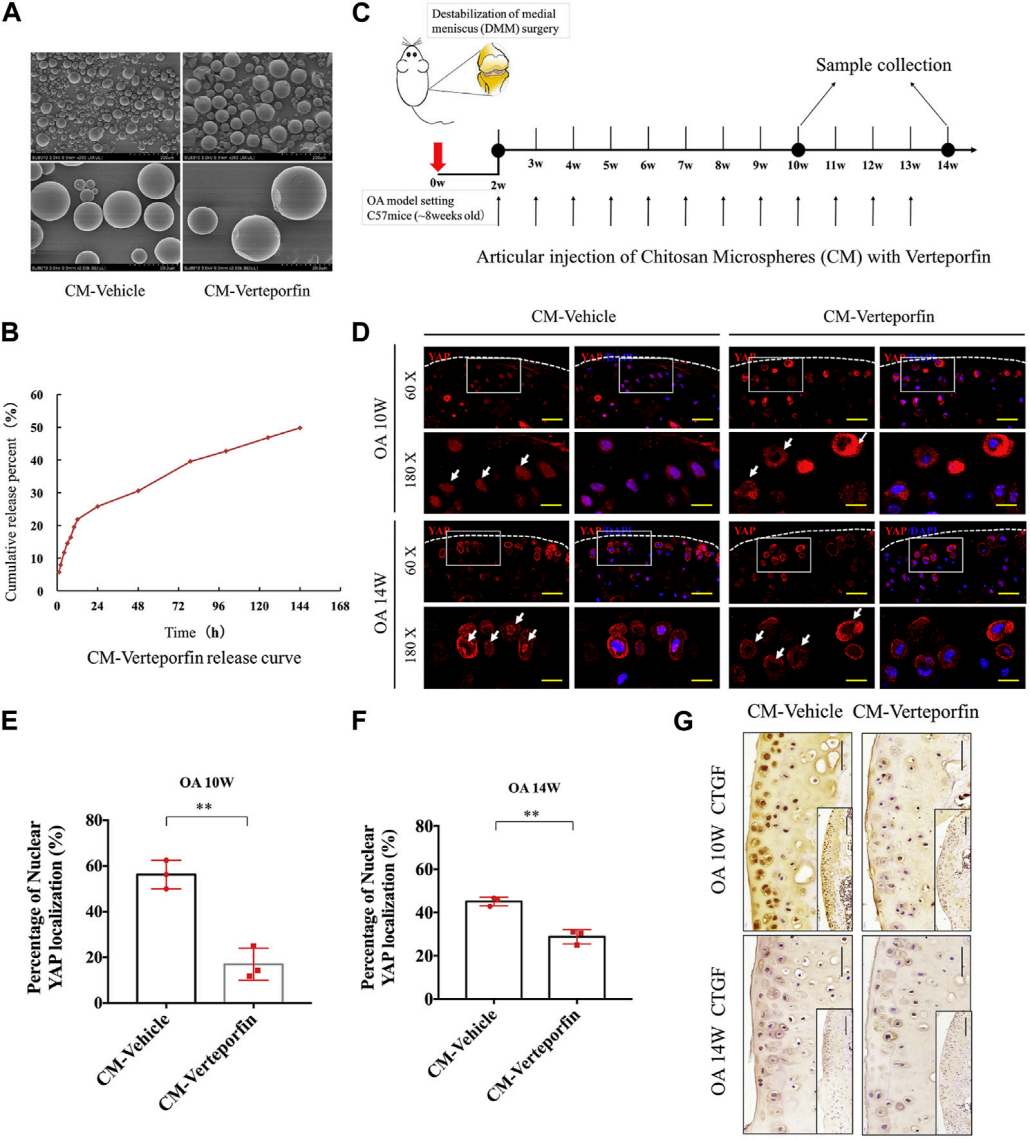
**(A)** Microscopic morphology of chitosan microspheres (CM) and verteporfin-loaded CM. **(B)** *In vitro* release results of CMs loaded with verteporfin. **(C)** Schematic of DDS used to treat OA model of mice. **(D)** Immunofluorescence images of articular cartilage sections of OA mice in each experimental group. **(E, F)** Percentage of nuclear YAP localization in the treated groups at various times following surgery. **(G)** Immunohistochemical results in cartilage sections of OA mice at various times following surgery. Reproduced with permission from ref (Zhang et al., 2020). Copyright 2020 Elsevier Ltd.

Chitosan (CS) is a natural polymer material with biodegradability, biocompatibility, and mucoadhesion. It can be used as an excellent drug carrier material. Microspheres prepared from chitosan can improve drug stability, enhance efficacy, and minimize systemic adverse effects. Zhu et al. (2015) explored the Rac1’s function and mechanistic pathways in OA chondrocyte pathology and OA development. They developed a potential OA therapeutic strategy using polymers to encapsulate and release Rac1 inhibitors to modulate its activity. Specifically, the anterior cruciate ligament transection was used to create a mice OA model, and CS microspheres encapsulated with the Rac1 inhibitor NSC23766 were encapsulated within hyaluronic acid (HA). Then, the OA knee joint received an injection of the mixed system. The results demonstrated that the drug-loaded microsphere system was able to release the inhibitor continuously while lubricating the joints (Figure 1). To reduce the side effects associated with long-term oral administration of lornoxicam in KOA patients, Abd-Allah group prepared CS microspheres coated with clonoxicam based on ionotropic-gelation technique with tripolyphosphate acting as a crosslinker. Compared with lornoxicam solution, drug-loaded microspheres showed long-term *in vivo* anti-inflammatory effects in the monosodium iodoacetate (MIA)-induced rat osteoarthritis model, significantly reducing the histology, inflammation, and biochemical parameters (Abd-Allah et al., 2016). Subsequently, Zhang et al. (2020) found that YAP (Yes-associated protein), a co-transcriptional factor, may be a mechanistic mediator of extracellular matrix stiffness and a therapeutic target of osteoarthritis. The results demonstrated that chitosan microspheres loaded with a selective inhibitor of YAP can target activity of subcellular YAP and slow down OA progression. These findings may provide additional information regarding their application in the future OA transformation (Figure 2).

A class of injectable and hydrolytically degradable heparin-based delivery systems has also been prepared for the delivery of tumor necrosis factor-α-stimulated gene 6 (TSG-6) to slow the progression of OA. Heparin is a highly-sulphated natural polysaccharide. It can be used as a biomaterial carrier to bind to various positively charged proteins. TSG-6 is a protein known as an inhibitor of plasmin, and plasmin degrades the extracellular matrix in OA joints. Moreover, research has demonstrated that heparin sulfation is a crucial regulator of the antiplasmin bioactivity of TSG-6. Animal experiments also verified that the MPs prepared in this study are expected to be effective for TSG-6 delivery in OA treatment. Analysis after 21 days shows that MPs loaded with TSG-6 reduce the damage to the cartilage after medial meniscal transection (MMT) (Tellier et al., 2018).

Gelatin is widely used in drug delivery systems because it has high biocompatibility, biodegradability, does not produce other by-products after degradation in the body, is non-immunogenic, and has the same components and biological properties as collagen. Park’s group has loaded anti-inflammatory cytokines in gelatin microspheres through ion complexation and chelation. Since proteolytic enzymes expressed in arthritis attacks can specifically degrade gelatin, the as-prepared bioreactive gelatin microspheres can achieve targeted release of anti-inflammatory cytokines in joints with OA. The experimental results showed that gelatin microspheres could significantly reduce chondrocyte inflammation (up to 80%). Thus, this catabolic response-synchronized on-demand delivery system is universally applicable for wound healing applications, especially in preventing OA inflammation-mediated cartilage damage (Park et al., 2020). Ratanavaraporn et al. combined gelatin with silk fibroin to develop composite drug-loaded microspheres loaded with curcumin, also induced by MIA, to create a rat OA injury model. After 8 weeks of minimally invasive injection treatment of drug-loaded microspheres, the cell destruction in the joint and synovial tissues was effectively delayed, and the radiological and histological grading of articular cartilage damage and synovial tissue alterations in experimental animals were similar to those in normal rats. The incorporation of silk fibroin effectively reduced the degradation rate of the microsphere matrix, and curcumin was able to achieve a longer sustained release in the joints and showed a prolonged anti-inflammatory effect (Ratanavaraporn et al., 2017).

### 2.2 Synthetic polymeric microparticle-based drug delivery systems

Poly(lactic-co-glycolic acid) (PLGA), as the most commonly utilized synthetic polymer for developing drug-delivery microsystems, is biocompatibility, biodegradability, non-toxicity, and non-immunogenicity, and most importantly, the material has adjustable mechanical and degradation properties and has FDA approval for human use. Many researchers have prepared various PLGA drug-loaded microparticles for OA treatment, and some microparticle formulations have entered the stage of clinical studies.

**FIGURE 3:**
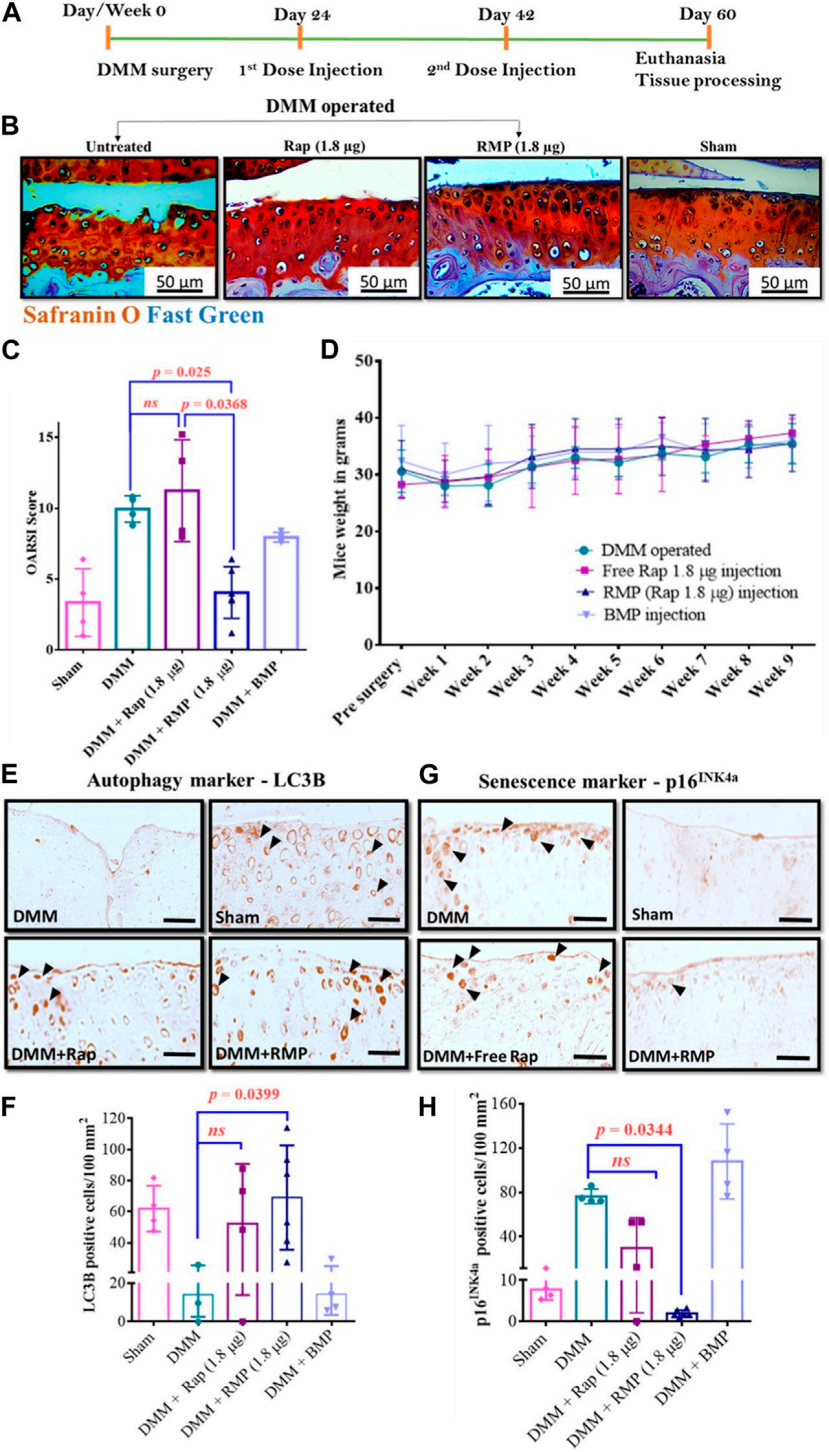
**(A)** Diagram illustrating the course of treatment. **(B)** Representative images of the mice knee joints’ medial tibial plateau (MTP) from various groups. **(C)** Results of OARSI scoring in mouse. **(D)** Changes in mice body weight over time following different treatments. Representative LC3B IHC-staining images **(E)** and number of LC3B-positive cells per unit area **(F)**. Representative p16INK4a IHC-staining images **(G)** and number of p16INK4a-positive cells per unit area **(H)**. Reproduced with permission from ref (Dhanabalan et al., 2023). CC BY 4.0. Copyright 2023 The Author(s).

Gómez-Gaete’s group prepared MPs loaded with rhein with anti-inflammatory properties by emulsion-solvent evaporation technique to improve the bioavailability of rhein and its utility in OA therapy. The morphology, encapsulation efficiency, and release behavior of drug-loaded MPs were investigated in a preliminary study, in addition to the evaluation of the *in vitro* cytotoxicity of the formulation (Gómez-Gaete et al., 2017). More recently, the safety of sterilization procedures for microparticle formulations before use has been systematically investigated. The results showed that the sterilization process did not have any major impact on the relevant properties of the microspheres loaded with drugs and no change in the *in vitro* release curve. In conclusion, gamma radiation is a suitable sterilization method, and intra-articular delivery of microparticle formulations also offers a promising therapeutic option for patients suffering from chronic joint disease (Avendaño-Godoy et al., 2023). Dhanabalan et al. (Dhanabalan et al., 2020; Dhanabalan et al., 2023) also developed MPs encapsulating rapamycin using PLGA as a carrier. The microsphere preparation is non-toxic and biocompatible and can achieve the sustained-release of rapamycin over several weeks. The MPs can effectively induce autophagy, prevent cellular senescence in human chondrocytes, and reduce inflammatory markers. Moreover, rapamycin MPs treatment maintained sulphated glycosaminoglycan (sGAG) production in 3D cultures under genotoxicity and oxidative stress conditions. This biomaterial-based rapamycin delivery system is expected to be further clinically translated as a therapeutic approach that meets patient requirements and provides new insight into aging prevention and autophagy activation therapies for treating trauma-induced OA via sustained-release formulations (Figure 3).

**FIGURE 4:**
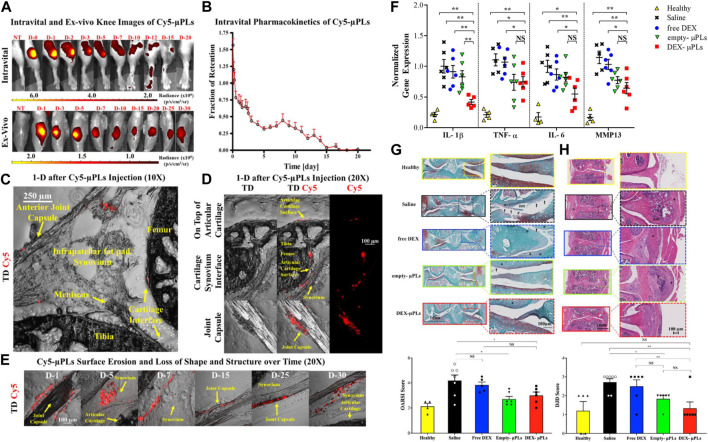
**(A)** Representative intravital images and *ex vivo* images of the knee joints of PTOA mouse injected with Cy5-μPLs. **(B)** *In vivo* retention fraction of Cy5-μPLs. **(C)** Anatomically labeled sagittal sections of mouse knee joint. **(D)** Distribution of Cy5-μPLs. **(E)** Imaging of Cy5-μPLs distribution after injection into the knee joint at various times. **(F)** Expression results of related cytokines. **(G)** Representative safranin-O stained images of tibial and femoral articular surfaces. **(H)** Representative H&E staining images of PTOA joints. Reproduced with permission from ref (Di Francesco et al., 2021). CC BY 4.0. Copyright 2021 The Author(s).

OA can also be managed with IA injections of steroid drugs examples include triamcinolone acetonide and dexamethasone for short-term pain relief. Bodick’s group (Bodick et al., 2018) developed a PLGA microsphere preparation coated with triamcinolone acetonide (TA), and IA injection of microspheres in experimental dogs can significantly prolong the release period of TA and cause mild foreign body reaction (FBR). Stefani et al. (2020) prepared a class of PLGA microsphere-agarose implants embedded with dexamethasone (DEX), which can continuously deliver low doses of DEX into the joint for at least 99 days. Chondroprotection observed *in vitro* in the context of IL-1-induced degradation and enhanced functional outcomes *in vivo*, and be able to optimize the IA delivery strategy of DEX to improve cartilage graft survival and functionality. Francesco’s research group (Di Francesco et al., 2021) developed a microscale PLGA plate loaded with dexamethasone. Microplates (μPLs) have tunable geometry and mechanical properties and can be deposited in joints for sustained release of DEX. Anti-inflammatory molecules were continuously released over 1 month in a biologically relevant confined volume and were detected to reduce the expression of inflammatory gene. Following administration of drug-laden μPLs to a mice model of knee overload injury (posttraumatic osteoarthritis, PTOA), a significant reduction in load-induced histological changes was observed. This innovative study also provides the proof-of-concept for the role of Shape-Defined μPLs in preventing joint degeneration associated with PTOA (Figure 4). Liang et al. (2021) also developed a novel PLGA-based microsphere formulation by oil-in-water emulsion and solvent evaporation method, which was loaded with mometasone furoate and was capable of stable, sustained release for more than 1 month. The release of the preparation in PBS was affected by the erosion of PLGA and proceeded by a non-Fick diffusion mechanism. The relevant research results demonstrated the long-term effectiveness and good biosecurity of the preparation. Hence, the delivery system has considerable application potential in the field of treatment of knee arthritis.

**FIGURE 5:**
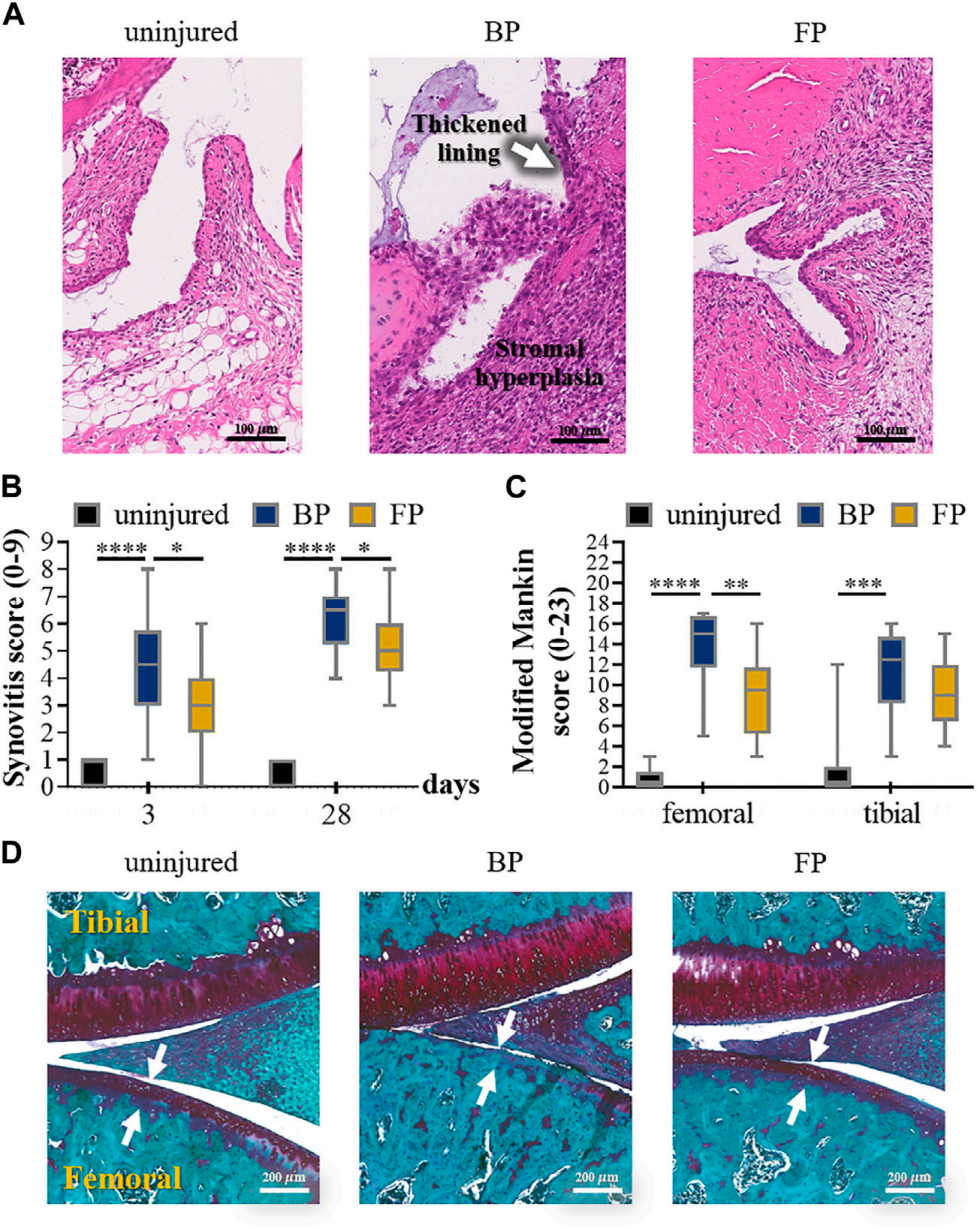
**(A)** Representative images of HE-stained synovial tissue on the third day after the injury to anterior cruciate ligament (ACL). **(B)** 2 blinded observers evaluated the individual synovitis score results according to the experimental procedures. **(C)** Results of the semi-quantitative assessment of the severity of osteoarthritis by modified Mankin score. **(D)** The individual osteoarthritis scores using S.E. 4 weeks after injury. Reproduced with permission from ref (Sangsuwan et al., 2022). Copyright 2022 Acta Materialia Inc.

**FIGURE 6:**
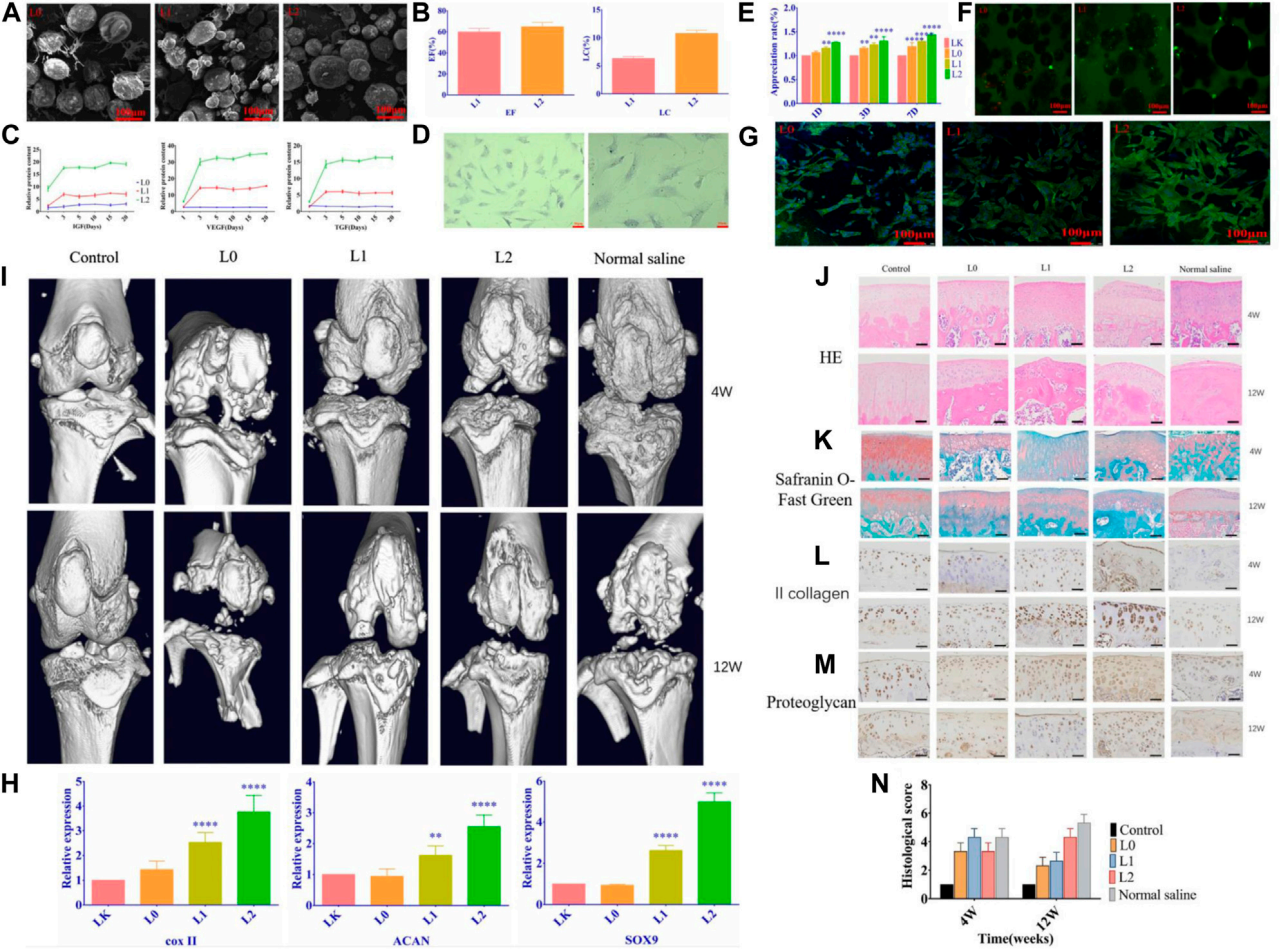
**(A)** SEM images of the microspheres. **(B)** Loading rate and encapsulation rate of the microspheres. **(C)** The release curve of related growth factors. **(D)** Optical micrograph of chondrocytes. **(E)**Proliferation of chondrocytes co-cultured with microspheres. **(F)** The percentage of viable cells after having treated with different groups of microspheres. **(G)** Cellular changes in microsphere-cultured cells. **(H)** Each group’s relative expression of chondrogenic genes. **(I)** Micro-CT scan images of the effect of microspheres on bone and articular cartilage damage. HE staining. **(J)** and Safranin-O fast green staining **(K)** results images. **(L)** Type II collagen staining results **(M)** Chondrocyte proteoglycan staining results. **(N)** Histological scoring of the cartilage according to OARSI. Reproduced with permission from ref (Li et al., 2021). CC BY 4.0. Copyright 2021 The Author(s).

Flavopiridol is capable of making progress in the treatment of PTOA by inhibiting cyclin-dependent kinase 9 (CDK9). For prolongation of Flavopiridol retention time in joints, Sangsuwan et al. (2022) prepared corresponding microsphere preparations by encapsulating Flavopiridol with PLGA microparticles as a strategy to reduce PTOA-related inflammation by inhibiting CDK9. The characterization results of drug-loaded microparticles showed that the microspheres had good injectability, low phagocytic potential, and cytotoxic potential and could maintain drug activity in a high-temperature environment. Flavopiridol microparticles subsequently exhibited the expected slow-release behavior in the PTOA rat knee injury model. The joint inflammation index (matrix metalloproteinase, MMP) was significantly reduced after 3 days of injection, and the drug-loaded microparticles could reduce the severity of PTOA after 28 days of injury. Thus, the drug-loaded microparticle formulation developed in this study was verified as a promising biomaterial platform as a potential therapeutic option for PTOA (Figure 5). Platelet lysate is a bioactive substance released from platelets and has a variety of biological functions. It contains various cell growth factors, cytokines, and many proteins required for cell proliferation. Li et al. (2021) as well prepared chitosan/gelatin/PLGA three-phase hybrid microspheres loaded with superactive platelet lysate (sPL) for the prevention and treatment of OA. The sPL-loaded microparticle formulation markedly increased proliferation of chondrocyte, reduced cell necrosis, and resulted in a smooth cartilage surface when administered to animal models while increasing cartilage integrity. This biofactor delivery platform has the potential for efficient and non-invasive repair of articular cartilage in OA (Figure 6).

Further, Bodick’s group conducted a series of preclinical studies on the PLGA microsphere preparation (FX006) loaded with TA based on previous studies to evaluate its clinical transformation potential. First, the team recruited 81 KOA patients intending to compare the pharmacokinetics of IA injections of TA sustained-release microsphere formulation (FX006) with that of crystalline suspension (TAcs) at the diseased joints of the patients. The results of the clinical research showed that a single FX006 injection significantly prolonged TA retention time in the joint and reduced peak plasma levels, thereby attenuating the systemic toxic effects of TA in humans (Kraus et al., 2018). This was followed by a preclinical study of the analgesic effects of different doses of FX006, which showed that FX006 provided long-term symptomatic relief, that 32 mg FX006 provided a higher therapeutic benefit, and that the preparation’s good safety profile was initially confirmed (Conaghan et al., 2018a). The symptom benefit and biosafety of FX006 were subsequently evaluated compared with saline placebo and TAcs. Compared to saline placebo, FX006 afforded significant and clinically meaningful pain relief in the primary endpoint. However, the improvement in osteoarthritis pain was insignificant with FX006 compared with TAcs (Conaghan et al., 2018b). About 30% of patients with type II diabetes suffer from KOA. Because of the glucose-raising effect of steroids, Bodick’s team recently carried out another clinical randomized, double-blind study to investigate the changes in blood glucose levels of patients after IA injection of FX006 and TAcs. The trial results indicated that IA injection of FX006 could be administered with minimal interference with glycemic control in patients with OA and type II diabetes (Russell et al., 2018). The above-mentioned series of preclinical research results also show that the FX006 preparation has good potential for clinical application in treating KOA.

In addition, a number of researchers have selected other types of synthetic polymers as delivery matrix platforms. Ho et al. (2019) used spray-drying technology to incorporate triamcinolone acetonide microcrystals into PLGA/PLA polymer microspheres (MSs). The proposed drug-loaded MSs can extend the period of drug retention in the rat joints while loading the systemic exposure of TA is significantly reduced (<50%). MSs also have excellent physicochemical stability, and the drug crystallinity and release profile do not change within 1 year. Similarly, polymeric particles coated with ketorolac were recently developed by Wongrakpanich et al. (2023) for treating OA using PLGA/PLA and their mixtures as carrier materials. A matrix composed of two homopolymers is more convenient to provide tailored drug release kinetics and improved physicochemical properties. As an alternative for OA patients, the drug-loaded microparticles can improve patient compliance and reduce treatment costs. Another research team prepared PLGA/PCL microspheres by solvent emulsification and evaporation method for prolonging the release of aceclofenac through parenteral administration. *In vivo* research findings demonstrated that the microspheres showed significant anti-inflammatory activity and were also promising in OA treatment (Kaur et al., 2017).

Abou-ElNour et al. (2019) copolymerized polyδ-decalactone (PDL) with methoxy poly(ethylene glycol) (mPEG) to form PEG-PDL with different molecular weights and subsequently co-blended with poly(propylene glycol) (PLA) to prepare novel composite particles loaded with triamcinolone acetonide. Afterward, the drug-loaded microparticles were administered to the rat knee osteoarthritis model. Compared with the drug suspension, the retention time of triamcinolone acetonide in the joint was longer, resulting in a more pronounced inhibition of inflammation. Sandker’s team developed a biodegradable microsphere formulation that can continuously deliver the anti-inflammatory drug tacrolimus into the joint based on the P(DLLA-PEG)-b-PLA multi-block copolymer. It is easy to adjust the release kinetics of the formulation by changing the ratio of hydrophilic and hydrophobic blocks. The drug can be released in the joint continuously for more than 1 month without causing systemic effects, and the excellent biocompatibility and local anti-inflammatory effect of the microsphere formulation were confirmed (Sandker et al., 2018).

Polyester amide (PEA) is a polymer based on aliphatic dicarboxylic acids, α-amino acids, and aliphatic α-ω diols (Castaldo et al., 1992). The presence of amino acid components in the structure makes it easy to be enzymatically broken down by proteolytic enzymes. Serine proteases exist in synovial fluid and are key components of the inflammatory response, which makes it possible for PEA-based DDSs to complete the bioreactive release of drugs along with the process of tissue inflammation (Nakano et al., 1999; Sharony et al., 2010). In recent years, Woike’s research group has developed a PEA-based polymer microsphere platform to load various therapeutic agents to expand the therapeutic options for KOA. In one of the studies, the group loaded celecoxib inside PEA microspheres to develop a safe DDS with self-regulating behavior. It has been verified that the degradation rate of PEA microspheres is higher in the inflammatory environment, while the celecoxib-loaded microspheres can significantly reduce the degradation rate of PEA, which indicates that the DDS does have autoregulatory behavior (Janssen et al., 2016). Subsequently, the effect of celecoxib polyester amide microspheres on disease relief and the optimal dose was further investigated in the rat OA model. No systemic and local adverse reactions were observed in the experimental group of rats. At the same time, cartilage histology was not affected (Tellegen et al., 2018). Thus, celecoxib PEA microspheres are expected to be a promising strategy for alleviating inflammation and pain in OA. In another study, TA was loaded into the PEA microsphere platform. TA microspheres can achieve sustained release over 60 days in PBS. Drug-loaded microspheres were retained for up to 70 days after intra-articular injection in OA, and synovial inflammation in diseased joints was significantly reduced with no effect on cartilage integrity. Longer pain relief and greater reduction in claudication were achieved than with TA-coated PLGA microspheres. In sum, the PEA microsphere platform demonstrated safety and efficacy when injected intra-articularly, making it an attractive potential OA treatment (Rudnik-Jansen et al., 2017; Rudnik-Jansen et al., 2019).

**FIGURE 7:**
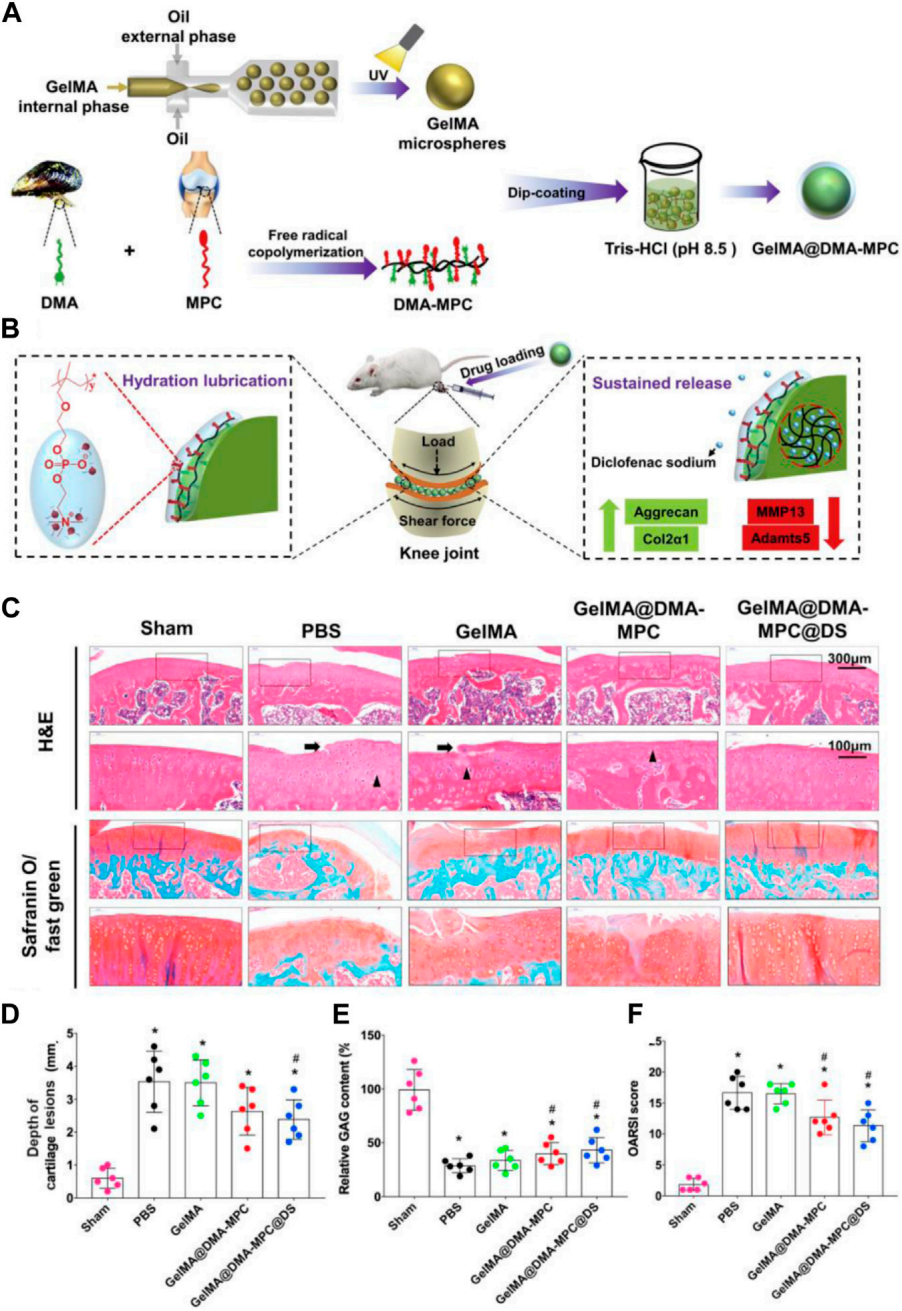
**(A)** Schematic illustration of GelMA@DMA-MPC microspheres preparation. **(B)** Delaying the OA progression using GelMA@DMA-MPC drug-loaded microspheres injected intra-articularly. **(C)** Representative images of rat cartilage sections with H&E and Safranin O-Fast Green staining (Arrows point to eroded fissures, triangles represent cloned tissue cellular structures). **(D)** The corresponding cartilage lesions depth, **(E)** relative GAG content and **(F)** OARSI score of rats’ articular cartilage. Reproduced with permission from ref (Han et al., 2021). CC BY-NC-ND 4.0 Copyright 2021 The Author(s).

Yang et al. (2020) prepared a type of polyampholyte modified methacrylate microspheres by using microfluidic technology. Among them, the grafting of poly(sulfobetaine methacrylate) provided the microspheres with stronger lubricity, lower degradation rate and sustained drug release properties. Subsequently, super-lubricating microspheres with excellent biocompatibility loaded with diclofenac sodium (DS) were further developed, which showed great potential in the osteoarthritis treatment by providing excellent hydrated lubrication and sustained drug release while protecting chondrocytes from inflammatory factor-induced degeneration *in vitro*. In a subsequent study, the research team selected 2-methacryloyloxyethyl phosphorylcholine (MPC) grafted methacrylate gelatin to prepare injectable hydrogel drug-loaded microspheres (GelMA@DMA-MPC@DS) with enhanced lubricity by photo-cross-linking, which confirmed that biocompatible functionalized microspheres provide significant therapeutic effects on OA development and represent a simple and promising technology for OA treatment (Han et al., 2021) (Figure 7).

### 2.3 Composite matrix microparticle-based drug delivery systems

Moreover, composite carriers between nanoparticles, liposomes, hydrogels, and microspheres can incorporate the advantages of various material forms, showing stronger plasticity and functionality than traditional single carriers, and the composite carrier platform is often loaded with two or more therapeutic agents to achieve more significant therapeutic effects. Thus, it is anticipated to be a promising novel topical drug delivery systems.

Yu et al. (2022) developed reactive oxygen species (ROS)-responsive hydrogel microspheres with cartilage-targeting. Specifically, ROS-responsive drug-loaded nanoparticles and type II collagen-targeting peptides were anchored in the hydrogel matrix by microfluidic technology, thereafter a polymerization process is implemented via UV light. The introduction of targeting peptides can achieve real targeted therapy, and the ROS-responsive nanoparticles react with the ROS in the cells and induce the drug to achieve a responsive and steady release, which can further reduce the inflammation and facilitate cartilage differentiation for achieving local and long-term therapeutic benefits after injection. Thus, it has important application prospects in OA treatment. Fang et al. (2022) developed a new microsphere-microcrystal-gel delivery system in which DEX-loaded microspheres were prepared from sodium alginate and hyaluronic acid. Moreover, another drug called celecoxib (CLX) microcrystals (CM) were fabricated using ultrasonication to enhance solubility and bioavailability. Finally, an injectable gel was prepared by cross-linking method to encapsulate both therapeutic agents. *In vitro*, the synergistic drug delivery system enabled sustained drug release and showed good biocompatibility and anti-inflammatory characteristics. *In vivo*, the release of inflammatory cytokines was downregulated, and cartilage matrix erosion and chondrocyte loss were significantly ameliorated. Therefore, this composite delivery platform offers a promising treatment to revolutionize traditional therapies for chronic joint diseases. Injectable degradable hydrogel microspheres with drug-loaded liposomes as a secondary structure have also been developed. The liposomes loaded with liquiritin (LQ) have a good effect on scavenging ROS in chondrocytes, and the chondroitin sulfate obtained from ChsMA gel degraded by hyaluronidase can also effectively fulfil its antioxidant activity. Thus, the composite delivery platform can provide dual antioxidant effects to eliminate ROS for OA therapy, and this novel delivery system also becomes a prospective option for treating osteoarthritis (He et al., 2022).

## 3 Conclusion and outlook

In the field of medicine, polymeric materials provide novel platforms for delivering drugs to target tissues. Novel formulations using polymers as drug delivery matrix have advantages over conventional formulations regarding prolonged drug residence time, improved curative effect, and enhanced patient compliance. There is currently a lack of effective and convenient treatments for OA, so developing new drug delivery systems is required for safe treatment. Intra-articular drug delivery strategies play an essential role in treating KOA, and different intra-articular drug delivery systems (IA DDSs) are being developed rapidly and have made notable progress. Desirable properties of IA DDSs for KOA treatment include excellent sustained and/or controlled release drug delivery, lubricating properties, and disease targeting. In this review, we searched, collected, and analyzed studies on applying different microsphere-based IA DDSs in preclinical/clinical trials (Table 1). Microsphere formulations are widely in use for the controlled delivery of therapeutic agents to enhance efficacy and reduce systemic drug toxicity. However, stability and scale-up of microsphere preparation are still issues to be resolved. In addition, the duration of efficacy, targeting properties, and the safety of the formulations still have major challenges.

**TABLE 1:** Drug-loaded microspheres DDSs with different polymer carriers for OA treatment.

Natural polymers				
Polymer carriers	Drug	Animal model	Outcome	Refs
Chitosan/Hyaluronic acid	Rac1 inhibitor NSC23766	OA model in mice	The preparation has a lubricating effect, can achieve sustained release of inhibitors, and can protect cartilage from damage and delay OA development	Zhu et al. (2015)
Chitosan	Lornoxicam	OA model in rat	It reduces inflammatory conditions and has long-lasting pharmacological effects	Abd-Allah et al. (2016)
Chitosan	Verteporfin	OA model in mice	The continuous release of Verteporfin can dramatically lower YAP activity in chondrocytes, effectively maintain cartilage homeostasis, and delay OA development	Zhang et al. (2020)
Heparin	Tumor necrosis factor-α-stimulated gene 6 (TSG-6)	Medial meniscal transection model in rat	Sulfated heparin can increase the biological activity of TSG-6, and IA injection of drug-loaded microspheres can lessen cartilage damage	Tellier et al. (2018)
Gelatin	IL-4; IL-10; IL-13	N/A	Drug-loaded microspheres are biologically responsive, prolong the residence time of anti-inflammatory cytokines, and significantly reduce inflammation	Park et al. (2020)
Gelatin/silk fibroin	Curcumin	OA model in rats	It delays cell destruction in joint and synovial tissues and exhibits long-lasting anti-inflammatory effects	Ratanavaraporn et al. (2017)
Synthetic polymers				
Poly (lactide-co-glycolide)(PLGA)	Rhein	N/A	Suitable for intra-articular administration in the treatment of OA after sterilization by gamma irradiation	Gómez-Gaete et al. (2017), Avendaño-Godoy et al. (2023)
PLGA	Rapamycin	OA model in mice	It can induce autophagy and prevent the aging of human chondrocytes, reducing cartilage damage and inflammation	Dhanabalan et al. (2020), Dhanabalan et al. (2023)
PLGA	Triamcinolone acetonide	Normal knee joint of the beagle	Extend drug retention time and reduce dosing frequency	Bodick et al. (2018)
PLGA	Triamcinolone acetonide	KOA patients	Extend the residence time of drugs in joints and lessen systemic side effects of patients. It has good biosafety and clinically provides long-term symptom relief	Conaghan et al. (2018a), Conaghan et al. (2018b), Kraus et al. (2018)
PLGA/Agarose	Dexamethasone	Adult mongrel dogs	It provides cartilage protection *in vitro* and improves function *in vivo*	Stefani et al. (2020)
PLGA	Dexamethasone	Posttraumatic osteoarthritis model in mice	Reduces the expression of inflammatory genes and comprehensively protects articular cartilage and broader joint structures	Di Francesco et al. (2021)
PLGA	Mometasone Furoate	Male Sprague Dawley rats	Stable and long-acting sustained release, good efficacy, and high safety *in vivo*	Liang et al. (2021)
PLGA	Flavopiridol	Posttraumatic osteoarthritis model in rat	Reduce inflammation and PTOA damage	Sangsuwan et al. (2022)
PLGA/Chitosan/Gelatin	superactive platelet lysate (sPL)	OA model in SD male rats	It increases chondrocyte proliferation, smoothes the cartilage surface, and increases cartilage integrity	Li et al. (2021)
PLGA/PLA	Triamcinolone acetonide	N/A	Extend drug retention time and reduce systemic exposure and dosing frequency	Ho et al. (2019)
PLGA/PLA	Ketorolac	N/A	Sustained and stable drug release	Wongrakpanich et al. (2023)
PLGA/PCL	Aceclofenac	Paw oedema model in female Sprague Dawley (SD) rats	Significant anti-inflammatory activity *in vivo*	Kaur et al. (2017)
PLA/PEG/poly-δ-decalactone (PDL)	Triamcinolone acetonide	KOA model in rats	Exhibits sustained anti-inflammatory effects	Abou-ElNour et al. (2019)
P(DLLA-PEG)-b-PLA	Tacrolimus	Horse joints	Excellent local anti-inflammatory ability and no systemic side effects	Sandker et al. (2018)
Polyester amide (PEA)	Celecoxib	OA model in rats	Reduce OA inflammation and pain	Janssen et al. (2016), Tellegen et al. (2018)
PEA	Triamcinolone acetonide	KOA model in rats	Synovial inflammation in diseased joints is significantly reduced	Rudnik-Jansen et al. (2017), Rudnik-Jansen et al. (2019)
Poly (dopamine methacrylamide-to-sulfobetaine methacrylate)/Gelatin	Diclofenac sodium	OA model in rats	It reduces osteophyte load and cartilage degradation and has therapeutic effects on osteoarthritis	Yang et al. (2020)

Multifunctional drug delivery systems with good biosafety and drug targeting are a prospective trend for the further development of IA DDSs. It is gaining attraction to combine different strategies to prepare DDSs for treating KOA. Nevertheless, synergistic effects between different strategies need to be taken into account as the strategies for DDSs become more complex. Generally, it is still necessary to further expand the research and application of polymers in KOA drug delivery systems to provide guidance for future clinical trials and enhance the long-term retention of drugs in the joints and diffusion to targeted tissues. At the same time, the benefits of novel therapies are meticulously balanced against their costs and potential risks.
